# Didelphys uterus: a successful induction of labor in a case of intrauterine fetal death near term

**DOI:** 10.1515/crpm-2021-0078

**Published:** 2022-02-23

**Authors:** Algeri Paola, D’Oria Patrizia, Toto Valentina, Fenili Paola, Ermito Santina, Bonalumi Silvia, Rinaldo Denise, Ciammella Massimo

**Affiliations:** Department of Obstetrics and Gynaecology, ASST Bergamo-est, Seriate, Bergamo, Italy; Complex Unit of Pathological Anatomy and Medical Genetics, San Paolo Hospital, Milan, Italy

**Keywords:** delivery, induction of labor, intrauterine death, Müllerian malformation, renal malformation

## Abstract

**Objectives:**

Müllerian anomalies are associated with infertility and worse pregnancy outcomes.

**Case presentation:**

A 34-years-old primigravida patient affected by didelphys uterus and type 2 diabetes mellitus was admitted at 36.4 weeks with intrauterine fetal death. Labor was induced with oral Mifepristone and vaginal Dinoprostone. She had an uneventful vaginal delivery.

**Conclusions:**

Pre-gestational evaluation should be recommended in each woman, in order to optimize clinical conditions in case of a chronic disease; moreover, if the patient is infertile Müllerian malformations should be excluded. In a didelphys uterus, the combination of Mifepristone and Dinoprostone could be a safe option for labor induction.

## Introduction

In the general population, 5.5–6.7% of the female population is affected by uterine malformations [1]. The first classification of these anomalies was reported in 1988 by The American Society for Reproduction Medicine [2], and revised in 2013 by European Society of Human Reproduction and Embryology-European Society for Gynaecological Endoscopy [3]. A correlation between renal anomaly and Müllerian anomalies was observed more frequently in infertile women, suggesting a link between these conditions [4].

Even when a pregnancy occurs, poorer outcomes were detected, in particular: spontaneous abortion, recurrent miscarriage, premature labor, caesarean delivery due to non-vertex fetal presentation, and decreased live births, compared to a normal uterus [4], [5], [6], [7], [8]. Didelphys uterus reported a 45% incidence of preterm birth.

Even though didelphys uterus is not an indication for caesarean delivery [9], [10], [11], a study reported that the risk of uterine rupture during labor (in case of vaginal birth after caesarean delivery) is 0.6% in normal uterus compared to 8% in Müllerian anomalies [12]. Therefore, there is a lack of information about uterine rupture in an unscarred uterus, a higher risk could be observed in labor, especially in case of induction.

To the best of our knowledge, there are no guidelines/studies that define which kind of induction could show lower risk of uterine rupture in the event of uterine malformations.

## Case presentation

**Figure 1:**
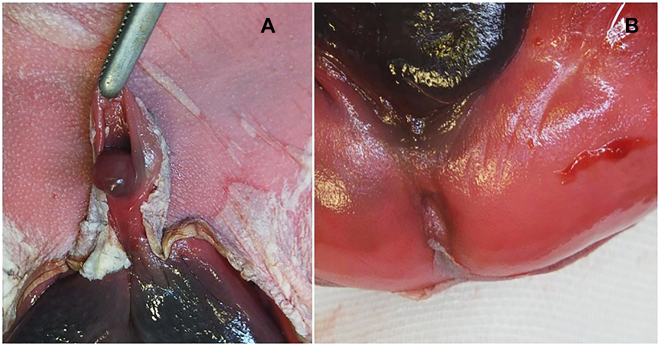
Macroscopic particular of neonatal authopsia. (A) Hypospadias. (B) Imperforate anus.

A 34 years old, non-Caucasian, primigravida booked at our Department at 13.2 weeks. Her mother was affected by diabetes mellitus. She reported a personal history of 7 years of infertility without any assessment and she was obese, with a body mass index (BMI) of 33. An ultrasound examination was performed: a didelphys uterus with a fetus located in the right horn was diagnosed; at the same time a right renal agenesis was detected. Diabetes mellitus type 2 was diagnosed and she began a therapy with insulin. The pregnancy was intensively monitored, with ultrasounds performed every two weeks in the first/second trimesters and weekly in the third one. She underwent regular diabetological evaluations with well controlled glucose levels by insulin therapy until 32nd week afterwards the dose was then implemented (Novorapid 10 UI at each meal and Levemir 18 UI after dinner). A scan during the 30-s week displayed a possible fetal hypospadias. At 34.3 weeks, the woman forgot to bring her glycaemic diary and missed the fetal biophysical score evaluation booked at 35 weeks. At 36.4 weeks, intrauterine fetal death was diagnosed. All tests performed were normal: COVID-19 research, infections, Kleihauer test, autoimmunity; standard and molecular array-CGH amniocentesis did not find any anomaly. A counselling was performed to the couple about the mode of delivery, offering vaginal one. All risks were elucidated and the possibility of failure of induction of labor was explained. However, considering the possible surgical complications of caesarean section and the patient’s wish to carry another pregnancy soon, we agree with the woman to induce labor. Vaginal evaluation before the induction found two cervixs both conservate and closed, with bishop score 0. Vagina was regular and no blind emivagina or septi were detected. We opted for oral Mifepristone 600 mg and, after 24 h, we continued with vaginal Dinoprostone 10 mg. After 10 h from Dinoprostone administration labor began, and within 3 h a male death neonate was delivered vaginally, without complications. Indirect signs of uterine rupture were strictly monitored. The baby’s weight was adequate for gestational age (2358 g). The autopsy examination detected hypospadias and imperforate anus (Figure 1A and B), without other malformations. Placental histological examination showed chronic ischemic alterations.

## Discussion

Considering the lack of information about the induction of labor in women with Müllerian anomalies, we decided to report our case in which we successfully used Mifepristone in association with Dinoprostone to induce third trimester abortive labor. Active labor lasted only 3 h. No maternal complications occurred.

FIGO 2017 guidelines suggested the administration of Mifepristone together with Misoprostol in case of intrauterine death. However, the possibility of uterine rupture in women with previous caesarean sections using Misoprostol for induction of labor should be considered [13]. Our patient had an unscarred uterus, but was diagnosed with a didelphys uterus, which could be a risk factor of uterine rupture itself [12]. Therefore, we preferred to use Dinoprostone, which has a safer profile, giving less tetanic uterine contractions.

We considered the possibility of a failed induction, and we informed the couple about that risk, but we opted for a vaginal delivery in order to avoid the risks of surgery on a didelphys uterus also taking into account the patient’s wish to get for another pregnancy soon. We also wanted to avoid the risk of uterine rupture (reported 8% in case of future pregnancy in a scarred didelphys uterus) [12].

In literature, few data are reported about the correct doses and kinds of drugs to induce labor in a didelphys uterus. Vaginal, oral or systemic prostaglandins are considered to be safe and effective to induce labor in standard conditions [13, 14]. The risk of uterine rupture or of failed cervical dilatation was observed and described in a case of pregnancy located into a rudimentary horn in didelphys uterus. In literature, a case report sustained that Misoprostol was not able to determine cervical modifications [15]; in another reported case, induction was performed with Dinoprostone without the achievement of labor (at caesarean section pregnancy was found in a rudimentary horn not communicant with the uterus) [15]. In a third case, the authors reported a failed induction of labor with no cervical dilation after the administration of the combination of vaginal and intramuscular PGE2 associated with PGF2a in a uterus with no communicant horn [14]. In contrast, didelphys uterus is reported to be associated with the possibility of vaginal delivery.

Our patient had a pregnancy complicated by a former undiagnosed didelphys uterus and by a poorly controlled diabetes mellitus with no pre-gestational evaluation. Her pregnancy was further complicated by the intrauterine fetal death near term, likely caused by the decompensated diabetes mellitus. As frequently reported in case of Müllerian anomalies, our patient was affected by infertility and renal anomaly [4]. Even if an ultrasound examination could fail the identification of Mullerian malformations, the diagnosis before pregnancy remains a challenge. The finding of these conditions during pregnancy or during labor, exposes the patient to an high risk of adverse obstetrical outcomes and to an higher risk of caesarean sections due to failure of the induction.

In conclusion, a pre-gestational evaluation should be recommended in each woman, in order to optimize clinical conditions in the case event of a chronic disease; moreover, in case of infertility, a gynaecological evaluation should be performed in order to exclude Müllerian malformations, considering the association between these conditions [4]. In this study we reported only one case but, considering the rarity of this condition, we decided to present it, to implement information about this theme. Our experience suggests that the association between Mifepristone and Dinoprostone could be a safe option in case of labor induction in a didelphys uterus. Our successful vaginal delivery should be further confirmed by other cases.
